# Hepatitis delta virus infection in Turkey: A meta-analysis of prevalence

**DOI:** 10.1016/j.ijregi.2024.02.003

**Published:** 2024-02-22

**Authors:** Mehlika Toy, Begüm Güler, Kayra Somay, Genco Gençdal, Cihan Yurdaydin

**Affiliations:** 1 Stanford University School of Medicine, Department of Surgery, Stanford, USA; 2 Koç University Medical School, Department of Gastroenterology & Hepatology, Istanbul, Turkey

**Keywords:** Delta hepatitis, Orphan disease, Turkey, Meta-analysis of prevalence

## Abstract

**Objectives:**

Hepatitis delta virus (HDV) infection has been granted orphan disease status by the US Food and Drug Administration and the European Medicines Agency owing to its rarity and relatively limited research and treatment options. Turkey is considered an endemic country for the virus. We aimed to provide a current and updated country- and region-specific HDV infection prevalence.

**Methods:**

In this meta-analysis, we searched databases, including MEDLINE, PUBMED, EMBASE, and UlakBim (Turkish Medical Index) published between January 1, 2006, and December 31, 2022. We included blood donor studies, outpatient clinic studies that comprised patients without cirrhosis, and inpatient clinical studies that comprised patients with cirrhosis. Turkey was divided into three regions: West, Central, and East Turkey.

**Results:**

After a systematic assessment, 41 studies were included. Using a random-effects model, the estimated HDV prevalence among hepatitis B surface antigen–positive blood donors, outpatient clinic, and inpatient clinic patients were 3.37% (confidence interval [CI] 1.99-6.11), 5.05% (CI 4.00-6.23), and 29.06% (CI 10.45-51.79), respectively. The HDV prevalence among outpatient clinic patients in Western, Central, and Eastern regions were 3.38% (CI 2.47-4.44), 2.15% (CI 1.37-3.09), and 9.81% (CI 6.61-13.55), respectively.

**Conclusions:**

East Turkey continues to have a high burden of HDV. Public health efforts, such as screening, should be targeted accordingly.

## Highlights

• Turkey is known to have a high burden of hepatitis B and D virus infections.
• Turkey is a country that serves as a bridge between Europe and Asia.
• The delta hepatitis prevalence among hepatitis B surface antigen–positive blood donors in Turkey is 3.37%.
• East Turkey continues to be a hot spot for delta hepatitis prevalence.
• The findings in this study are not confined to the border of Turkey alone.

## Introduction

Hepatitis delta virus (HDV) infection is recognized as the most severe form of viral hepatitis. HDV is a defective virus that requires the hepatitis B virus (HBV) to propagate and to cause disease in humans [1]. In a systemic review done in 2011, hepatitis B surface antigen (HBsAg) positivity was estimated to be around 4.57% in Turkey, with a large age and regional difference which ranged from 2.84% to 6.36% [2] and, according to a meta-analysis encompassing the years 1980 to 2005, the anti-HDV prevalence ranged from 4.8% to 27% among patients with chronic HBV infection without cirrhosis and from 20% to 46% for patients with cirrhosis [3]. A more recent study, the TURHEP field work study, using sound statistic methodology covering the whole country and screened around 5,533 people, reported a 4% HBV prevalence and a 2.8% anti-HDV prevalence among HBsAg-positive individuals [4].

HDV epidemiology has been described as occurring in three epidemiological patterns: (i) as an endemic disease occurring in a large proportion of chronic HBsAg-positive individuals, (ii) as an epidemic disease striking isolated HBsAg-positive communities, and (iii) as a disease occurring only in high-risk groups [5]. Turkey is a region that has a high burden of HDV infection and is considered an endemic country for the virus, occurring in large proportions of HBsAg-positive individuals. In this study, we aimed to provide a current and updated delta hepatitis prevalence in Turkey.

## Methodology

The systematic review of the studies conforms to the guidelines outlined by the Preferred Reporting Items for Systematic Reviews and Meta-Analyses (PRISMA) [6]. For English and/or Turkish language studies, the databases MEDLINE, PUBMED, EMBASE, and UlakBim (Turkish Medical Index) were searched using the following terms: “Hepatitis D [and] Turkey,” “Hepatitis delta prevalence [and] Turkey.” All articles were reviewed and their corresponding reference lists were inspected to identify additional material that had not been detected initially. These were later either retrieved by a new electronic search or searched manually. The period for the meta-analysis ranges from 2006 to 2022. The first-round review criteria for selection of studies included were the availability of explicit data on the country region, setting (e.g. hospital), study period, number of subjects studied and number of subjects positive for HBsAg and anti-HDV, or stated crude prevalence. We took measures to detect and extract overlapping reports on the same study population or its fraction. These measures included an analysis of the study period, sample size, centers where studies were performed, and author names.

Once a study was included, the following data were extracted and entered into tables: study region, study type (general or hospital based), total number of persons studied/tested, number of persons positive for HBsAg, number of persons tested positive for anti-HDV, and year of publication. Three of the authors (MT, BG, and KS) checked all studies used in the analysis. The same method was used by including region as a predictor to obtain region-specific estimates. Data were analyzed using the MetaXL version 5.3 software, an add-in for meta-analysis in Microsoft Excel for Windows (https://www.epigear.com/index_files/metaxl.html).

To account for the potential sources of heterogeneity in the data, we used two types of statistical models: a random-effects model and a quality effects model. The quality effects model allowed us to examine the effects of study quality on the prevalence estimate independent of other covariates, whereas the random-effects model accounted for both within-study and between-study heterogeneity and provided an estimate of the average effect size across studies. The quality effects model uses a quality score (Appendix Table 1), which is essential as a safe-guard score (risk of bias instrument). We scored each study that was used for the meta-analysis (Appendix Table 2).

The random-effects model, based on the DerSimonian–Laird method, which calculates the variability within and between studies, was applied to estimate the pooled prevalence and 95% confidence intervals (CIs). It was chosen over the fixed-effects model because it takes inherent study-to-study variability into account. The MetaXL software also allowed us to account for study quality in the meta-analysis [7]. The results are presented graphically in a forest plot.

Definitions that were used for the studied populations were the following:

1. Blood donors and general population (serosurvey studies), general population that were screened for anti-HDV antibodies,
2. Outpatient clinic studies, studies that reported prevalence among patients with hepatitis B e antigen (HBeAg)–negative chronic hepatitis B (CHB) infection and HBeAg-negative (without cirrhosis) CHB, and
3. Inpatient clinic studies, studies that report prevalence among patients with cirrhosis diagnosed clinically or by biopsy.

When studies reported inpatient and outpatient prevalence in one study, we took that study in both analyses, separating the data reported into inpatient and outpatient categories. This study also reports the pooled prevalence by region. The following pooling of regions were done: region A: Marmara and Aegean region representing Western Turkey; region B: Black Sea, Central Anatolia, and Mediterranean region, representing Central Turkey; region C: eastern and southeastern region, representing Eastern Turkey, based on several factors such as geography, population size, and socioeconomic status. Because of the small number of available studies, we only estimated the pooled prevalence for outpatient studies.

## Results

**Figure 1:**
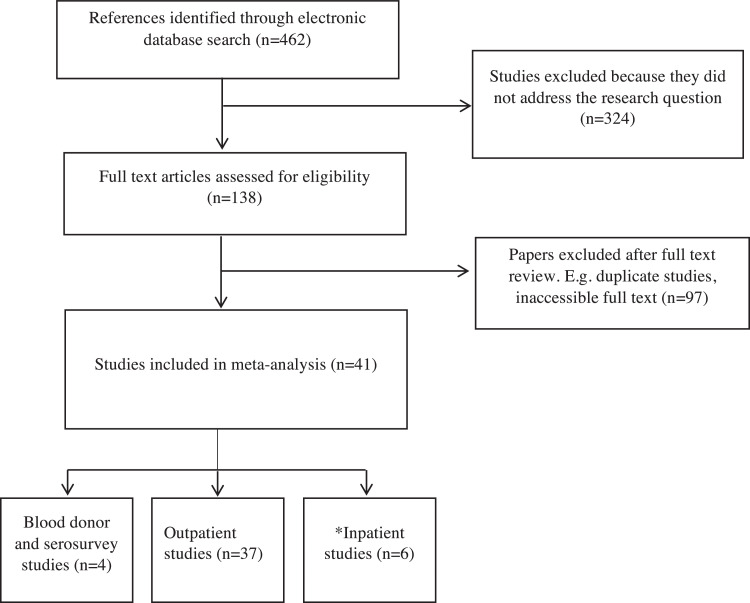
Flow diagram (selection strategy) of selected studies. *Because some studies reported blood donor, inpatient, and outpatient prevalence data, we used the same study and separated that for the sub-groups.

The results of the search strategy and final distribution of the studies are shown in Figure 1. The electronic search identified 277 studies, and manual reference checking identified an additional 185 references. There were eventually four large studies for the blood donor and serosurvey studies, 37 studies reported outpatient prevalence, and six studies reported inpatient prevalence. Some studies reported inpatient and outpatient prevalence data, which we extracted and used accordingly. The included studies are referenced in Supplementary Appendix 1.

**Figure 2:**
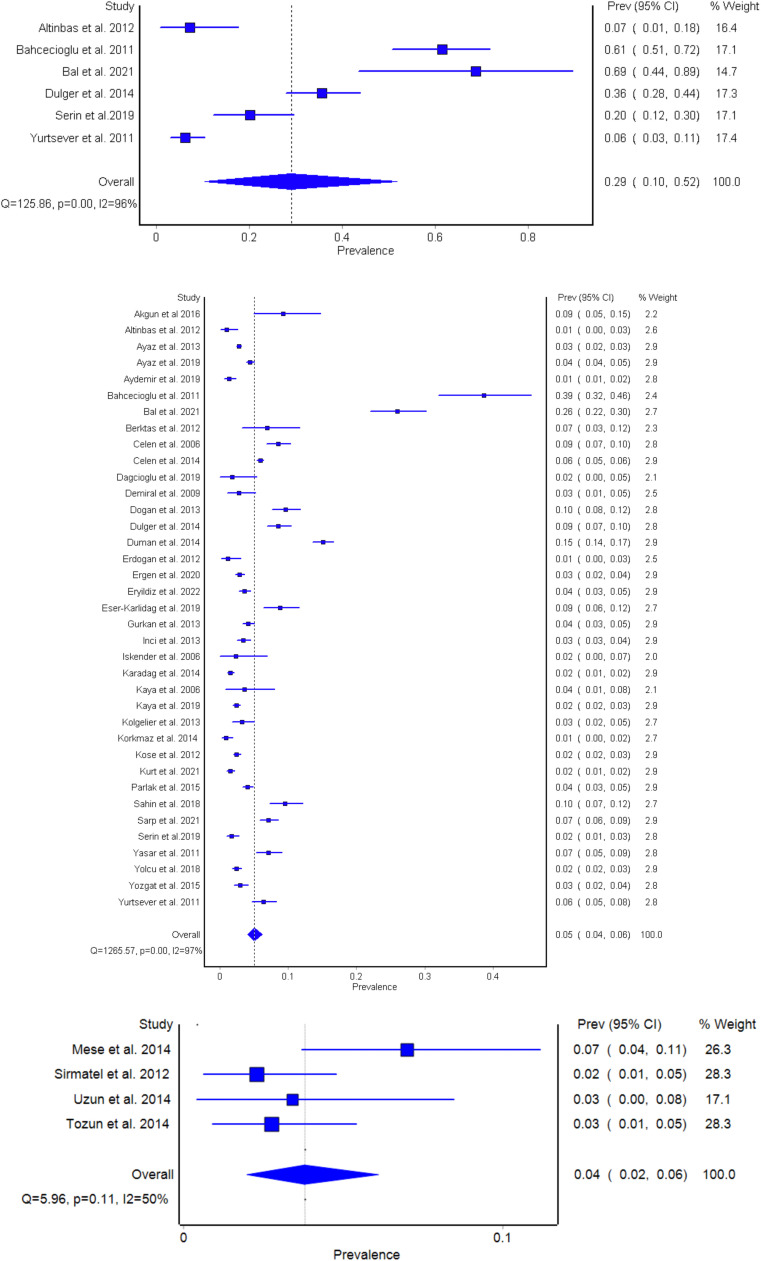
(a) Forest plot for outpatient studies (HBeAg-negative CHB infection and HBeAg-negative [non-cirrhotic] CHB). (b) Inpatient studies (patients with cirrhosis). (c) Blood donor and serosurvey studies. CI: confidence interval; CHB: chronic hepatitis B; HBeAg: hepatitis B e antigen.

The number of HBsAg-positive cases with cirrhosis was 1,083, with 274 having anti-delta positivity. The pooled prevalence of four studies was 29.06% (95% CI 10.45-51.79) (Figure 2a). The I^2^ is 96%, suggesting significant heterogeneity. The quality effects model estimates were comparable. Of these six studies, three were from Eastern Turkey [8,9], two were from Western Turkey [10,11] and one was from Central Turkey [12]. Interestingly, both studies from Western Turkey were from the very same hospital in Izmir [10,11]. The study by Serin et al. [10] that was published in 2019 reported an HDV prevalence of 20% among patients with cirrhosis with CHB, whereas in the study by Yurtsever et al. [11], which was published some 8 years earlier, HDV prevalence was merely 6%. In the study from Central Turkey, the HDV prevalence among patients with cirrhosis with CHB was 7% [10], whereas it had a pooled prevalence of 36% in patients in Eastern Turkey [11]. The quality effects model pooled prevalence results were comparable; the appendix contains the forest plot and quality score for the studies (Appendix Tables 3-6 and Appendix Figures 1 and 2).

**Figure 3:**
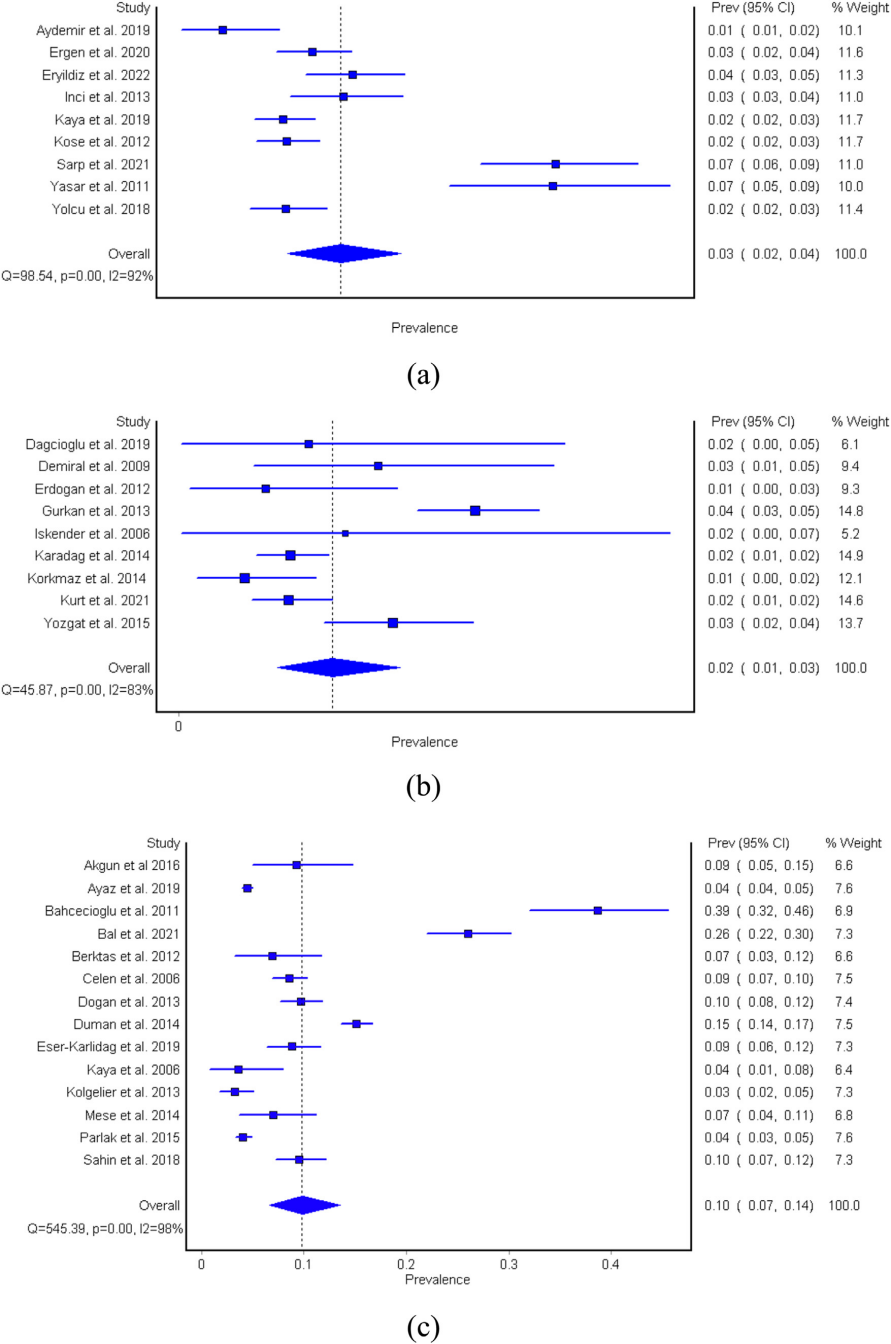
(a-c). Forest plots for the three regions. (a) Region A: Marmara and Aegean region, representing Western Turkey; (b) region B: Black Sea, Central Anatolia, and Mediterranean region, representing Central Turkey; (c) region C: Eastern and Southeastern region, representing Eastern Turkey. CI: confidence interval.

**Figure 4:**
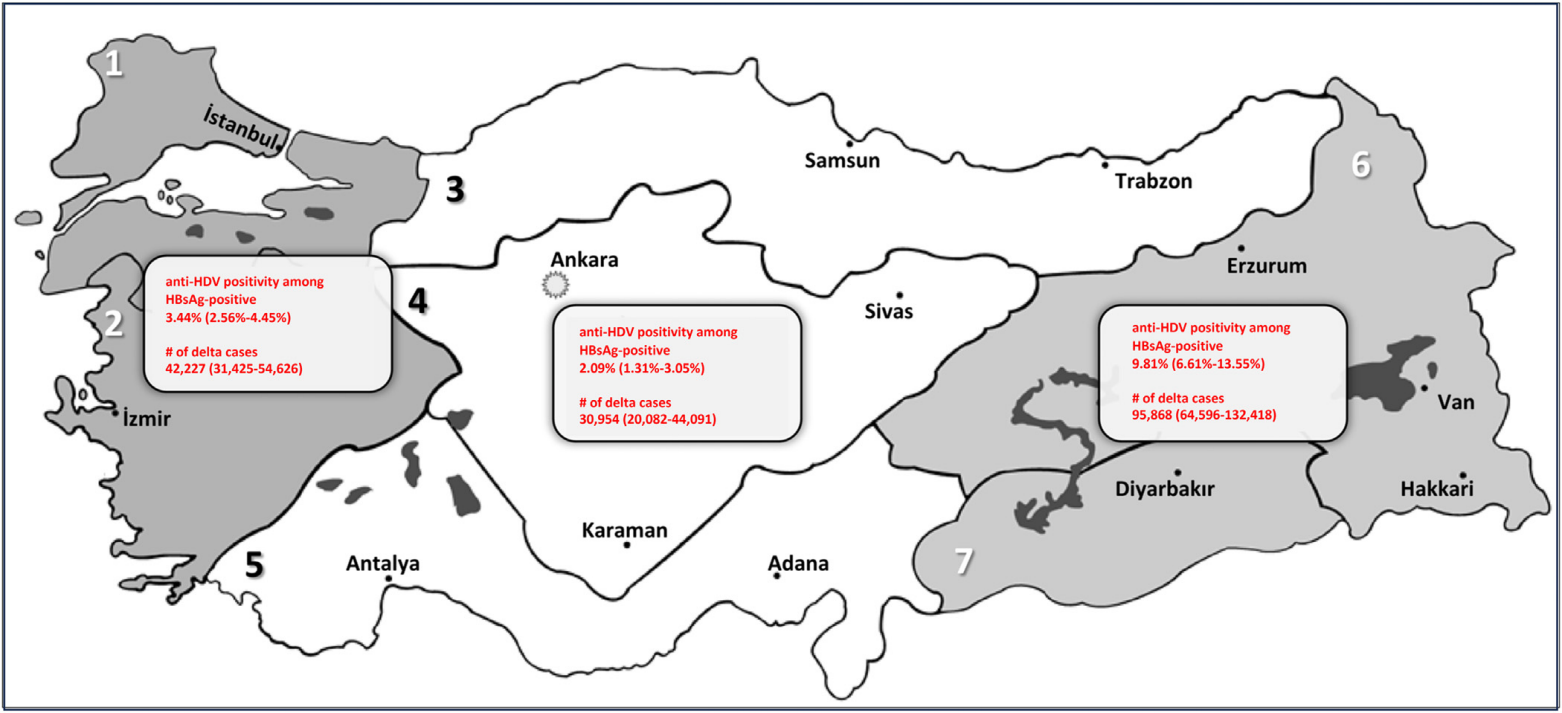
Map of Turkey according to regions, prevalence of anti-HDV, and the number of estimated delta cases for HBeAg-negative CHB infection and HBeAg-negative (non-cirrhotic) CHB only. *Map of Turkey according to regions; 1: Marmara region, 2: Aegean region, 3; Black Sea region, 4: Inner Anatolia region, 5: Mediterranean region, 6: Eastern Anatolia region, 7: Southeastern Anatolia region. Regions with similar socioeconomic status, HBsAg seroprevalence, and delta hepatitis prevalence are grouped as A (1 and 2), B (3, 4, and 5), and C (6 and 7). CHB: chronic hepatitis B; HBeAg: hepatitis B e antigen; HBsAg: hepatitis B surface antigen; HDV: hepatitis D virus.

In outpatients, the total number of HBsAg-positive patients in 37 studies was 58,085, with 2910 anti-delta positive cases without cirrhosis. The pooled prevalence was 5.05% (95% CI 4.00-6.23) (Figure 2b). The I^2^ is 97%, suggesting significant heterogeneity. The quality effects model estimates were comparable (Appendix Tables 7-10 and Figures 3 and 4).

The pooled prevalence for blood donor studies was 3.37% (95% CI 1.99-6.11) (Figure 2c). The I^2^ results suggest that there is only 50% heterogeneity among the studies. The quality effects model pooled prevalence results were comparable; the appendix contains the forest plot and quality score for the studies (Appendix Tables 11-14 and Appendix Figures 5 and 6). Three studies reported similar HDV prevalence rates of 2% to 4% in HBsAg-positive subjects [4,^13^,14], of which one was a serosurvey study [4], whereas the one outlier blood donor study reporting an HDV prevalence of 7% (of 186 HBsAg-positive cases) was from Eastern Turkey (city: Diyarbakir) [15]. However, in the former three studies, one study was also from Eastern Turkey (city: Şanlıurfa) but reported an HDV prevalence of 2.5% (of 194 HBsAg-positive cases) [13].

The pooled prevalence of HBsAg-positive patients without cirrhosis for regions A, B, and C were 3.38% (95% CI 2.47-4.44), 2.05% (95% CI 1.33-2.92), and 9.81% (95% CI 6.61-13.55), respectively. The I^2^ ranged from 81% to 97% (Figures 3a-3c), suggesting significant heterogeneity. The quality effects model estimates were comparable.

**Figure 5:**
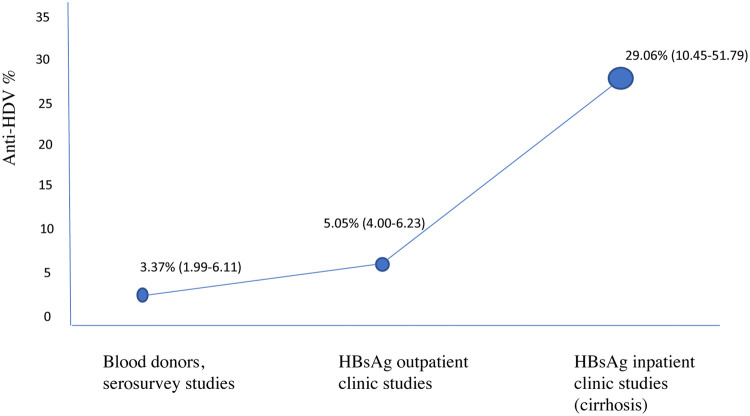
Anti-HDV prevalence in Turkey in various sub-groups. HBsAg: hepatitis B surface antigen; HDV: hepatitis D virus.

If one considers that Turkey's population is 85 million and we take the estimated blood donor prevalence for anti-delta positivity and multiply that with the 4% HBV prevalence, we will have an approximate 128,526 (67,484-207,202) cases of HDV among HBsAg-positive individuals and a general estimate prevalence of 0.15% in Turkey (Figure 4). If we take the prevalence estimates for each region and the prevalence estimate of HBV by region [2], the number slightly increases because regional studies are based on outpatient studies. The overall cases in each region would be the following: region A: 39,219 (22,637-67,398), region B: 14,009 (5,595-29,282), and region C: 95,868 (64,596-132,418). The anti-HDV prevalence in Turkey for each sub-group prevalence is shown in Figure 5.

## Discussion

This study is a meta-analysis of individual studies that were retrospective observations from single universities or in some cases multi-centered surveys. There is, to date, only one population-based field study, which was conducted by Tozun et al. [4] in 2009-2010, on the seroprevalence of HBV, hepatitis C virus, and HDV. This population-based study [4], comprising 5,533 screened individuals, reported a 4% HBV prevalence and a 2.8% prevalence among HBsAg-positive individuals for HDV, which is similar or slightly lower than our estimate of 3.37% in blood donors. In general, in Turkey and other neighboring countries such as Iran, the HBV prevalence data from blood donors are considered, to some extent, ambiguous because they may underestimate the HBV burden in the region because high-risk groups for HBV/HDV are rejected from blood donation without pre-transfusion HBsAg assessment [16,17]. Blood donation in Turkey is on a voluntary basis and assessment of high-risk groups is done through a detailed questionnaire before blood donation. Through this questionnaire, subjects who had used intravenous drugs; men who have sex with men; individuals who had paid for sexual intercourse; and patients with a history of HIV infection, hepatitis infection, or jaundice are rejected right away. Based on this approach, the HBV and HDV prevalence rates among blood donors are considered an underestimation of the real HBV and HDV burden. A recent representative example supporting this reasoning can be given from two studies [13,18]: one study of blood donors and one community-based study, performed in the Southeastern Turkish city of Şanlıurfa. Although in the former study, performed in 2006 in more than 19,000 blood donors, the HBsAg prevalence was found to be 2.9% [13]; in the latter, a community-based study conducted in 2008 using sound statistical cluster sampling methodology where the subjects for analysis were randomly selected from the catchment areas served by 19 different primary health care centers, the HBsAg prevalence was found to be 4.2% [18].

Nevertheless, blood donor studies have the advantage of providing consistent data based on a large number of subjects, which appear to be very suitable to follow the dynamic change of HBV prevalence in a particular country because sound population-based studies are difficult to perform financially and logistically. In using blood donor studies for the assessment of HBV and HDV prevalence, the underestimation factor we have discussed previously has to be considered. Hence, we decided to pool the data of blood donors with the one large population-based study. The rather acceptable heterogeneity of 50% between studies gives further support to our considerations. Because most of the prevalence studies did not report age-specific outcomes, it was not possible for us to run age-specific or age group–specific analyses. We understand that this might be a limitation of our study. Some of these studies reported the median age of the study population. The blood donor studies reported the median age to be around 40 years, whereas outpatient studies reported a median age of 44 years and inpatient studies reported a median age of around 48 years and older. This would suggest that blood donors are, in general, younger than the inpatient and outpatient HBsAg-positive patients, which may further contribute to the low prevalence rates among blood donors.

A previous systematic review [3] that looked at the prevalence between years 1980 and 2005 reported that between 1995 and 2005, the delta hepatitis prevalence in patients with chronic hepatitis B without cirrhosis were 27% and 12% in Southeast and Central Turkey, respectively. Because the previous meta-analysis reported the estimated prevalence until year 2005, the current study included studies that were published after 2006. Compared with our estimates, the seroprevalence of HDV among non-cirrhotic CHB decreased in Southeast and Central Turkey from 27% to 9.8% and 12% to 2%, respectively.

A recent systematic study was published by Uraz et al. [19]; using a very simple methodology, the authors calculated a mean, adding each prevalence from various studies, of which some studies included very few subjects. With this debatable methodology, Uraz et al. [19] reported that after 2010, the anti-HDV prevalence was 5.5% for all patients with HBV infection without cirrhosis (with an HDV prevalence estimate of 5.2% in inactive HBsAg carriers and of 6.9% in patients with CHB). Their estimate falls above the high range of our 95% CI (4.00-6.23) for all HBsAg-positive patients without cirrhosis (outpatient studies).

The results of the quality effects model and the random-effects model in our study were not substantially different from each other, suggesting that the impact of study quality on the prevalence estimate may not be a major source of heterogeneity in the data. However, it is still important to report the results of both models because it provides transparency in the analysis and allows a more comprehensive understanding of the sources of heterogeneity in the data. This overall impression, based on acceptable statistical know-how, is important because one may easily be confused by interpreting that the low prevalence estimates reported by studies among subjects without cirrhosis with CHB is the reported anti-HDV prevalence in inactive HBsAg carriers (HBeAg-negative CHB infection with the new terminology as suggested by the latest European Association for the Study of the Liver [EASL] guidelines on the management of HBV) [20] or in patients without cirrhosis with CHB. Even in small-scale studies performed in tertiary centers, there appears to be little effort to differentiate between these two conditions, which may be attributed to a lack of non-invasive instruments, such as the routine use of quantitative HBsAg measurements or the lack of wide-spread availability of transient elastography. On the other hand, it may also suggest that milder forms of chronic HDV may be increasing [21], [22], [23], [24]. The lack of a robust and cheap biomarker availability at every clinical setting to differentiate HBeAg-negative CHB infection from HBeAg-negative (non-cirrhotic) CHB is likely one reason why there is significant heterogeneity between outpatient studies. Another reason is the variability in HDV prevalence based on geographic origin, as can be seen in Figures 3a-c and Figure 4. In Turkey, the HDV screening rate in HBsAg-positive individuals is yet to be studied. A study by Papatheodoridis et al. [25] that studied the HDV screening and prevalence rates in HBsAg-positive patients seen in tertiary hospitals throughout Greece reports that screening rates vary widely among Greek liver clinics, ranging between 85 and 88%, with an approximate screening rate of 40% in tertiary centers. Another HDV screening study done in Barcelona by Palom et al. [26] reported a 7.6% screening rate and provided evidence to how reflex testing increases the number of anti-HDV positive cases detected.

Even studies performed in hospitalized patients with cirrhosis displayed significant heterogeneity. This heterogeneity could be explained, to some extent, by the geographic origin of the studies—higher HDV prevalence in East Turkey versus lower prevalence in West and Central Turkey. However, we encountered two studies from West Turkey in which the anti-HDV prevalence in patients with cirrhosis was reported in one study as 6% [11] and in another study performed around 8 years later from the very same hospital as 20% [10] of patients with CHB. Talking to the authors of the study, we learned that the latter study comprised sicker patients in general; in the earlier study, all patients had compensated liver disease, whereas in the latter study, around 20% had decompensated cirrhosis (Vatansever, personal communication). Thus, although this detail explained the controversial data in the two studies, it also highlights the dilemma of performing reliable large-scale epidemiological studies based on published material [27].

One of the strengths of this meta-analysis is that it includes studies written in Turkish to overcome language bias; thus, this analysis provides information that would be accessible to researchers and policymakers from other countries in the world. In recent years, migration from high endemic areas plays an important role in the increasing HBsAg-positive cases in western countries [28], [29], [30]. The importance of immigration-related HBV increase is also the case in Turkey, which is highlighted in a study performed in refugee children from Syria, where a striking 4.2% HBsAg positivity was reported [31]. Furthermore, although international immigrants constituted 2% of the total population in Turkey in 2000, this increased to 6% in 2017 [30]. A limitation of our study is that, unfortunately, none of the prevalence studies took immigrants into consideration, which the most important risk group after 2000 for HBV and HDV [30] in Turkey. This is a very important deficiency of studies performed after 2006, in particular, after 2011, mass immigration from Syria has started to be followed in recent years by migration from Afghanistan. The prevalence of HBsAg positivity among migrants needs to be further studied in Turkey.

In conclusion, this study highlights the challenges in attaining reliable data from individual prevalence studies and pooling those for a meta-analysis even at a country level. However, these meta-analyses with critical assessment of data are still very important in the context of developing health strategies in a particular country. Overall, the data of this meta-analysis suggest that HDV continues to be an important problem, particularly, in Eastern Turkey, despite evidence of a decrease in its endemicity in this country, and can be used for developing health policy implementations. Finally, in the era of mass migration, testing for anti-HDV in all HBsAg-positive individuals and in those found positive for HDV RNA, as is also recommended by the recent EASL HDV guidelines [32], should be a strategy not confined to HDV endemic countries but also to industrial countries where HDV-induced liver disease is considered an orphan disease.

## Declarations of competing interest

The authors have no competing interest to declare.

## Appendix Supplementary materials

Image, application 1
