# Supplementary material for: Hepatitis delta virus infection in Turkey: A meta-analysis of prevalence
Source: IJID Reg. 2024 Feb 22;10:228–34. doi: 10.1016/j.ijregi.2024.02.003 (PMC10912444; doi:10.1016/j.ijregi.2024.02.003)
Supplement: Supplementary file 1 [file mmc1.docx]

Appendix: **Hepatitis Delta Virus Infection in Turkey: A Meta-Analysis of Prevalence**

**Contents**

[Appendix 1: Included studies 2](#_Toc148463567)

[Appendix Table 1. Health States Quality Index Variables 6](#_Toc148463568)

[Appendix Table 2. Quality Effects Score for Each Study 7](#_Toc148463569)

[Appendix Table 3. Random Effects Model Output for Inpatient Studies 9](#_Toc148463570)

[Appendix Table 4. Random Effects Model Sensitivity for Inpatient Studies 10](#_Toc148463571)

[Appendix Table 5. Quality Effects Model for Inpatient Studies 11](#_Toc148463572)

[Appendix Table 6. Quality Effects Model Sensitivity for Inpatient Studies 12](#_Toc148463573)

[Appendix Table 7. Random Effects Model Output for Outpatient Studies 13](#_Toc148463574)

[Appendix Table 8. Random Effects Model Sensitivity for Outpatient Studies 16](#_Toc148463575)

[Appendix Table 9. Quality Effects Model Output for Outpatient Studies 18](#_Toc148463576)

[Appendix Table 10. Quality Effects Model Sensitivity Output for Outpatient Studies 21](#_Toc148463577)

[Appendix Table 11. Random Effects Model Output for Blood Donor and Serosurvey Studies 23](#_Toc148463578)

[Appendix Table 12. Sensitivity Analysis per Study for Blood Donor and Serosurvey Studies 24](#_Toc148463579)

[Appendix Table 13. Quality Effects Model Output for Blood Donor and Serosurvey Studies 25](#_Toc148463580)

[Appendix Table 14. Quality Effects Model Blood Donor and Serosurvey Studies Sensitivity 26](#_Toc148463581)

[Appendix Figure 1: DOI plot for the inpatient studies to assess the impact of individual studies on the overall estimate of effect size 27](#_Toc148463582)

[Appendix Figure 2: Funnel plot for the inpatient studies to assess the presence of publication bias 28](#_Toc148463583)

[Appendix Figure 3: DOI plot for the outpatient studies to assess the impact of individual studies on the overall estimate of effect size 29](#_Toc148463584)

[Appendix Figure 2: Funnel plot for the inpatient studies to assess the presence of publication bias 30](#_Toc148463585)

[Appendix Figure 5: DOI plot for the blood donor and serosurvey studies to assess the impact of individual studies on the overall estimate of effect size 31](#_Toc148463586)

[Appendix Figure 6: Funnel plot for the blood donor and serosurvey studies to assess the presence of publication bias 32](#_Toc148463587)

# Appendix 1: Included studies

**Blood donor and serosurvey studies**

Tozun, N., et al., Seroprevalence of hepatitis B and C virus infections and risk factors in Turkey: a fieldwork TURHEP study. Clin Microbiol Infect, 2015. 21(11): p. 1020-6.

Mese, S., et al., Seroprevalence of serum HBsAg positivity and hepatitis delta virus infection among blood donors in Southeastern Turkey. La Clinica Terapeutica, 2014. 165(2).

Sirmatel, F., et al., Saglikli kan donorlerinde hepatit B virus, hepatits C virus ve hepatitis D virus seroprevalansi. Viral Hepatitis Journal, 2012. 18(1): p. 19-22.

Uzun, B., et al., Evaluation of hepatitis delta virus (HDV) infection in blood donors in western Turkey. Transfus Apher Sci, 2014. 50(3): p. 388-91.

**Outpatient studies**

Akgun, S., et al., HBV-DNA, HCV-RNA ve HDV-RNA birlikteliginin degerlindirilmesi: 2 yillik retro spektif calisma. Ortadogu Tip Dergisi, 2016. 8(4): p. 186-189.

Altinbas, A., et al., HBsAg pozitif hastalarda delta hepatit seropozitiflik sikligi. Cumhuriyet Medical Journal, 2012. 34(1): p. 56-59.

Ayaz, C., et al., Epidemiology and Risk Factors of Hepatitis Delta Infection in Turkey. J Pure Appl Microbiol, 2013. 7(4): p. 2809-2813.

Ayaz, C. and T. Sari, Kronik delta hepatitli hastalarimizin tedavi sonuclari. Ortadogu Tip Dergisi, 2019. 11(1): p. 73-77.

Aydemir, O., et al., Hepatit B virusu ile infekte hastalarda delta hepatit pozitifligi; Koinfeksiyon mu, superinfeksiyon mu? Flora Infeksiyon Hastaliklari ve Klinik Mikrobiyoloji Dergisi, 2019. 24(2): p. 131-135.

Bahcecioglu, IH., et al., Prevalence of hepatitis delta virus (HDV) infection in chronic hepatitis B patients in eastern Turkey: still a serious problem to consider. Journal of Viral Hepatitis, 2011. 18: p. 518-524.

Bal T. Siirt Ilinde Yuksek Hepatit Delta Antikoru Sikligi. Journal of Duzce University Health Sciences Institute, 2021. 11(1): p. 45-49.

Berktas, M., et al., HBV-DNA Pozitif Olgularda HDV-RNA Sikligi. Viral Hepatit Dergisi, 2012. 18(1): p. 34-36.

Celen, M.K., et al., Anti-hepatitis delta virus seroprevalence and risk factors in patients with hepatitis B in Southeast Turkey. Saudi Med J, 2006. 27(5): p. 617-20.

Celen, M.K., et al., The characteristics of patients with chronic hepatitis B in Turkey. Med Glas (Zenica), 2014. 11(1): p. 94-98.

Dagcioglu, Y., et al., Tokat ili kronik hepatit B enfeksiyonlu hastalarda Tork Teno virus ve Hepatit D virus arastirilmasi. Turkish Journal of Clinics and Laboratory, 2019. 10: p. 314-318.

Demirdal, T., N. Demirturk, and Z. Asci, Afyonkarahisar ilinde hepatit delta virusu seroprevalansi. Viral Hepatit Dergisi, 2009. 14(3): p. 104-107.

Dogan, M., et al., Kronik hepatit B enfeksiyonlu hastalarda anti-HDV ve HDAg prevalansi. Dicle Tip Dergisi, 2013. 40: p. 50-53.

Dulger, A.C., et al., High prevalence of chronic hepatitis D virus infection in Eastern Turkey: urbanization of the disease. Arch Med Sci, 2016. 12(2): p. 415-20.

Duman, Y., M.S. Tekerekoglu, and S. Ay, Seroprevalence of HBsAg, anti-HBs, anti-HDV and HDVAg in Inonu University Medical Faculty Hospital, 2012. Medicine Science, 2014. 3: p. 982-990.

Erdogan, T., HBsAg pozitif, belirtisiz kronik hepatit B virus tasiyici hastalarin uzun donem degerlendirilmesi, in Infectious Diseases. 2012, Eskisehir Osmangazi Universitesi Tip Fakultesi: Eskisehir. p. 69.

Ergen, P., F. Yilmaz Karadag, and O. Aydin, Investigating the prevalence of hepatitis delta and assessment of treatment response. Viral Hepatit Dergisi, 2020. 26: p. 135-140.

Eryildiz, C., et al., Retrospective evaluation of hepatitis delta virus seroprevalence in HBsAg-positive patients. Turk Mikrobiyol Cemiy Derg, 2022. 52(3): p. 232-239.

Eser-Karlidag, G., Prevalence of hepatitis delta in chronic hepatitis B patients Klimik Derg, 2019. 32(3): p. 281-4.

Gurkan, Y., et al., Ankara Numune Eğitim ve Araştırma Hastanesi’ne 2010-2013 Yılları Arasında Başvuran Hastaların HBsAg ve Anti-HDV Seroprevalanslarının Değerlendirilmesi. Viral Hepatitis Journal, 2013. 19(3): p. 148-51.

Inci, A., M. Fincanci, and C. Muderrisoglu, Investigation of anti-hepatitis delta virus and anti-hepatitis C virus in patients with hepatitis B virus infection Istanbul Med J, 2013. 14: p. 109-11.

Iskender, G., et al., Seroprevalence of anti-hdv antibody in HBsAg positive patients. Acta Oncologica Turcica, 2006. 39: p. 99-100.

Karadag, A., et al., Defining the delta virus positivity in hepatitis B virus infections. Viral Hepatit Journal, 2014. 20(2): p. 64-66.

Kaya, S., Poliklinigimizde izlenen kronik hepatit B virus infeksiyonlu hastalarda anti-HDV sikligi. Viral Hepatit Dergisi, 2006. 11(3): p. 154-157.

Kaya, S., et al., Evaluation of hepatitis D virus serology results of Izmir Katip Celebi university Ataturk training and research hospital. J Immunol Clin Microbiol, 2019. 4(3): p. 91-96.

Kolgelier, S., N. Demir-Aktug, and S. Ozcimen, Seropositivity of delta hepatitis in HBsAg positive patients in Adiyaman Province. Viral Hepatit Dergisi, 2013. 19(1): p. 8-10.

Korkmaz, P., et al., Seropositivity of delta hepatitis in HBsAg positive patients in Eskisehir Province. Viral Hepatit Dergisi, 2014. 20(2): p. 72-74.

Kose, S., et al., Study on seroprevalence of hepatitis delta in a regional hospital in western Turkey. J Infect Dev Ctries, 2012. 6(11): p. 782-785.

Kurt, E.K., et al., Investigating hepatitis C, D and HIV prevalence in cases with positive hepatitis B virus antigen in a tertiary hospital and examining anti-HDV positive cases. Viral Hepatit Dergisi, 2021. 27(1): p. 31-35.

Parlak, E., et al., Assessment of patients with hepatitis D. Viral Hepatit Dergisi, 2015. 21(3): p. 80-84.

Sahin, A., et al., Anti-HDV seroprevalence among patients with previous HBV infection. North Clin Istanb, 2018. 5(2): p. 132-138.

Sarp, T.Z., et al., Retrospective evaluation of hepatitis delta virus infection seroprevalence. CBU-SBED, 2021. 8(3): p. 495-502.

Serin, A. and S. Vatansever, Seroprevalence of delta hepatitis in chronic hepatitis B patients: single center study. F.U.Med.J.Health.Sci, 2019. 33(3): p. 137-141.

Yasar, K.K., F. Pehlivanoglu, and G. Sengoz, Sekiz aylik donemde laboratuvarimizda saptanan hepatit B ve hepatiti D seroprevalansi. Viral Hepatit Dergisi, 2011. 17(1): p. 22-26.

Yolcu, A., et al., Frequency of hepatitis delta virus in hepatitis B surface-antigen positive patients. Viral Hepatit Dergisi, 2019. 25(1): p. 14-18.

Yozgat, A., et al., Delta hepatitis frequency in chronic hepatitis B patients: single center retrospective study. Ortadogu medical journal, 2015. 7(1): p. 12-15.

Yurtsever, S., et al., The prevalence and clinical significance of delta antibody in hepatitis B virus infection. Viral Hepatit Dergisi, 2011. 17(2): p. 69-73.

**Inpatient Studies**

Altinbas, A., et al., HBsAg pozitif hastalarda delta hepatit seropozitiflik sikligi. Cumhuriyet Medical Journal, 2012. 34(1): p. 56-59.

Bahcecioglu, IH., et al., Prevalence of hepatitis delta virus (HDV) infection in chronic hepatitis B patients in eastern Turkey: still a serious problem to consider. Journal of Viral Hepatitis, 2011. 18: p. 518-524.

Bal T. Siirt Ilinde Yuksek Hepatit Delta Antikoru Sikligi. Journal of Duzce University Health Sciences Institute, 2021. 11(1): p. 45-49.

Dulger, A.C., et al., High prevalence of chronic hepatitis D virus infection in Eastern Turkey: urbanization of the disease. Arch Med Sci, 2016. 12(2): p. 415-20.

Serin, A. and S. Vatansever, Seroprevalence of delta hepatitis in chronic hepatitis B patients: single center study. F.U.Med.J.Health.Sci, 2019. 33(3): p. 137-141.

Yurtsever, S., et al., The prevalence and clinical significance of delta antibody in hepatitis B virus infection. Viral Hepatit Dergisi, 2011. 17(2): p. 69-73.

# Appendix Table 1. Health States Quality Index Variables

| **Q1. Were the target population and the observation period well defined?**  Yes = 1  No = 2 |
| --- |
| **Q2. Diagnostic criteria**  Use of diagnostic system reported (DSM,ICD,RDC) = 1  Own system/symptoms described/no system/not specified = 0 |
| **Q3. Method of case ascertainment**  Community survey/multiple institutions = 2  Inpatient/inpatients and outpatients/case registers = 1  Not specified = 0 |
| **Q4. Administration of measurement protocol**  Administered interview = 3  Systematic casenote review = 2  Chart diagnosis/case records = 1  Not specified = 0 |
| **Q5. Catchment Area**  Broadly representative (national or multi-site survey) = 2  Small area/not representative (single community, single university) = 1  Convenience sampling/other (primary care sample/treatment group) = 0 |
| **Q6. Prevalence measure**  Point prevalence (e.g. one month) = 2  12-month prevalence = 1  Lifetime prevalence = 0 |

# Appendix Table 2. Quality Effects Score for Each Study

| **Study** | **Q1** | **Q2** | **Q3** | **Q4** | **Q5** | **Q6** | **Total Score (max 11)** | **Max score in this group of studies=10** | **Qi** |
| --- | --- | --- | --- | --- | --- | --- | --- | --- | --- |
| Akgun et al 2016 | 1 | 1 | 1 | 1 | 1 | 2 | 7 | 7/10= | 0.70 |
| Altinbas et al. 2012 | 1 | 1 | 1 | 1 | 1 | 2 | 7 | 7/10= | 0.70 |
| Ayaz et al. 2013 | 1 | 1 | 1 | 1 | 1 | 2 | 7 | 7/10= | 0.70 |
| Ayaz et al. 2019 | 1 | 1 | 2 | 2 | 2 | 2 | 10 | 10/10= | 1.00 |
| Aydemir et al. 2019 | 1 | 1 | 1 | 1 | 0 | 2 | 6 | 6/10= | 0.60 |
| Bahcecioglu et al. 2011 | 1 | 1 | 1 | 1 | 0 | 2 | 6 | 6/10= | 0.60 |
| Bal et al. 2021 | 1 | 1 | 1 | 1 | 0 | 2 | 6 | 6/10= | 0.60 |
| Berktas et al. 2012 | 1 | 1 | 1 | 1 | 1 | 2 | 7 | 7/10= | 0.70 |
| Celen et al. 2006 | 1 | 1 | 2 | 2 | 2 | 2 | 10 | 10/10= | 1.00 |
| Celen et al. 2014 | 1 | 1 | 2 | 2 | 2 | 2 | 10 | 10/10= | 1.00 |
| Dagcioglu et al. 2019 | 1 | 0 | 1 | 1 | 1 | 2 | 6 | 6/10= | 0.60 |
| Demiral et al. 2009 | 1 | 1 | 1 | 1 | 1 | 2 | 7 | 7/10= | 0.70 |
| Dogan et al. 2013 | 1 | 0 | 1 | 1 | 1 | 2 | 6 | 6/10= | 0.60 |
| Dulger et al. 2014 | 1 | 0 | 1 | 1 | 1 | 2 | 6 | 6/10= | 0.60 |
| Duman et al. 2014 | 1 | 1 | 1 | 1 | 1 | 2 | 7 | 7/10= | 0.70 |
| Erdogan et al. 2012 | 1 | 2 | 1 | 2 | 1 | 2 | 9 | 9/10= | 0.90 |
| Ergen et al. 2020 | 1 | 1 | 1 | 1 | 0 | 2 | 6 | 6/10= | 0.60 |
| Eryildiz et al. 2022 | 1 | 1 | 2 | 2 | 2 | 2 | 10 | 10/10= | 1.00 |
| Eser-Karlidag et al. 2019 | 1 | 1 | 1 | 1 | 1 | 2 | 7 | 7/10= | 0.70 |
| Gurkan et al. 2013 | 1 | 1 | 1 | 1 | 0 | 2 | 6 | 6/10= | 0.60 |
| Inci et al. 2013 | 1 | 1 | 1 | 1 | 0 | 2 | 6 | 6/10= | 0.60 |
| Iskender et al. 2006 | 1 | 0 | 1 | 1 | 0 | 2 | 5 | 5/10= | 0.50 |
| Karadag et al. 2014 | 1 | 1 | 1 | 1 | 1 | 2 | 7 | 7/10= | 0.70 |
| Kaya et al. 2006 | 1 | 1 | 1 | 1 | 1 | 2 | 7 | 7/10= | 0.70 |
| Kaya et al. 2019 | 1 | 1 | 1 | 1 | 1 | 2 | 7 | 7/10= | 0.70 |
| Kolgelier et al. 2013 | 1 | 1 | 1 | 1 | 0 | 2 | 6 | 6/10= | 0.60 |
| Korkmaz et al. 2014 | 1 | 1 | 1 | 1 | 1 | 2 | 7 | 7/10= | 0.70 |
| Kose et al. 2012 | 1 | 0 | 1 | 1 | 1 | 2 | 6 | 6/10= | 0.60 |
| Kurt et al. 2021 | 1 | 1 | 1 | 1 | 1 | 2 | 7 | 7/10= | 0.70 |
| Mese et al. 2014 | 1 | 0 | 2 | 2 | 2 | 2 | 9 | 9/10= | 0.90 |
| Parlak et al. 2015 | 1 | 1 | 1 | 1 | 1 | 2 | 7 | 7/10= | 0.70 |
| Sahin et al. 2018 | 1 | 0 | 1 | 1 | 1 | 2 | 6 | 6/10= | 0.60 |
| Sarp et al. 2021 | 1 | 1 | 1 | 1 | 1 | 2 | 7 | 7/10= | 0.70 |
| Serin et al.2019 | 1 | 1 | 1 | 1 | 1 | 2 | 7 | 7/10= | 0.70 |
| Sirmatel et al. 2012 | 1 | 1 | 2 | 2 | 2 | 2 | 10 | 10/10= | 1.00 |
| Tozun et al. 2014 | 1 | 1 | 2 | 2 | 2 | 2 | 10 | 10/10= | 1.00 |
| Uzun et al. 2014 | 1 | 1 | 1 | 1 | 1 | 2 | 7 | 7/10= | 0.70 |
| Yasar et al. 2011 | 1 | 1 | 1 | 1 | 1 | 2 | 7 | 7/10= | 0.70 |
| Yolcu et al. 2018 | 1 | 1 | 1 | 1 | 0 | 2 | 6 | 6/10= | 0.60 |
| Yozgat et al. 2015 | 1 | 1 | 1 | 1 | 1 | 2 | 7 | 7/10= | 0.70 |
| Yurtsever et al. 2011 | 1 | 1 | 1 | 1 | 0 | 2 | 6 | 6/10= | 0.60 |

# Appendix Table 3. Random Effects Model Output for Inpatient Studies

| **Random effects results** |  |  |  |  |  |  |
| --- | --- | --- | --- | --- | --- | --- |
|  |  |  |  |  |  |  |
| **Study** | Prevalence | LCI 95% | HCI 95% | weight (%) | HBsAg-Positive | delta positive |
| **Altinbas et al. 2012** | 0.073170732 | 0.009684359 | 0.177271796 | 16.40407332 | 41 | 3 |
| **Bahcecioglu et al. 2011** | 0.614457831 | 0.506928016 | 0.7167238 | 17.06416609 | 199 | 77 |
| **Bal et al. 2021** | 0.6875 | 0.436070278 | 0.894820878 | 14.69209988 | 446 | 116 |
| **Dulger et al. 2014** | 0.357142857 | 0.27961766 | 0.438572839 | 17.34393421 | 140 | 50 |
| **Serin et al.2019** | 0.202380952 | 0.122599333 | 0.295695326 | 17.07219922 | 84 | 17 |
| **Yurtsever et al. 2011** | 0.063583815 | 0.031406684 | 0.10543772 | 17.42352729 | 173 | 11 |
|  |  |  |  |  |  |  |
| **Pooled** | 0.290674426 | 0.104595208 | 0.51794881 | 100 |  |  |
| **Statistics** |  |  |  |  |  |  |
| **I-squared** | 96.02734036 | 93.50621945 | 97.56967078 |  |  |  |
| **Cochran's Q** | 125.8602662 |  |  |  |  |  |
| **Chi2, p** | 0 |  |  |  |  |  |
| **tau2** | 0.289228447 |  |  |  |  |  |

# Appendix Table 4. Random Effects Model Sensitivity for Inpatient Studies

| **Random effects model sensitivity** |  |  |  |  |  |  |  |
| --- | --- | --- | --- | --- | --- | --- | --- |
|  |  |  |  |  |  |  |  |
| **Study** | Pooled Prevalence | LCI 95% | HCI 95% | Cochran Q | I 2 | I 2 LCI 95% | I 2 HCI 95% |
| **Altinbas et al. 2012** | 0.3450 | 0.1215 | 0.6069 | 116.9133 | 96.5787 | 94.2272 | 97.9723 |
| **Bahcecioglu et al. 2011** | 0.2276 | 0.0751 | 0.4249 | 67.5130 | 94.0752 | 89.0171 | 96.8038 |
| **Bal et al. 2021** | 0.2276 | 0.0550 | 0.4598 | 112.0169 | 96.4291 | 93.9330 | 97.8983 |
| **Dulger et al. 2014** | 0.2747 | 0.0517 | 0.5694 | 113.7277 | 96.4828 | 94.0390 | 97.9248 |
| **Serin et al.2019** | 0.3098 | 0.0772 | 0.6000 | 125.0748 | 96.8019 | 94.6619 | 98.0840 |
| **Yurtsever et al. 2011** | 0.3563 | 0.1652 | 0.5724 | 59.2376 | 93.2475 | 87.1789 | 96.4437 |

# Appendix Table 5. Quality Effects Model for Inpatient Studies

| **Quality effects model** |  |  |  |  |
| --- | --- | --- | --- | --- |
|  |  |  |  |  |
| **Study** | Prevalence | LCI 95% | HCI 95% | weight (%) |
| **Altinbas et al. 2012** | 0.0732 | 0.0097 | 0.1773 | 9.5726 |
| **Bahcecioglu et al. 2011** | 0.6145 | 0.5069 | 0.7167 | 14.8718 |
| **Bal et al. 2021** | 0.6875 | 0.4361 | 0.8948 | 4.9430 |
| **Dulger et al. 2014** | 0.3571 | 0.2796 | 0.4386 | 23.9194 |
| **Serin et al.2019** | 0.2024 | 0.1226 | 0.2957 | 17.5356 |
| **Yurtsever et al. 2011** | 0.0636 | 0.0314 | 0.1054 | 29.1575 |
|  |  |  |  |  |
| **Pooled** | 0.2331 | 0.0518 | 0.4781 | 100 |
| **Statistics** |  |  |  |  |
| **I-squared** | 96.0273 | 93.5062 | 97.5697 |  |
| **Cochran's Q** | 125.8602662 |  |  |  |
| **Chi2, p** | 0 |  |  |  |
| **Q-Index** | 10.51587302 |  |  |  |

# Appendix Table 6. Quality Effects Model Sensitivity for Inpatient Studies

| **Quality effects sensitivity** |  |  |  |  |  |  |  |
| --- | --- | --- | --- | --- | --- | --- | --- |
|  |  |  |  |  |  |  |  |
| **Study** | Pooled Prevalence | LCI 95% | HCI 95% | Cochran Q | I 2 | I 2 LCI 95% | I 2 HCI 95% |
| **Altinbas et al. 2012** | 0.2548 | 0.0479 | 0.5335 | 116.9133 | 96.5787 | 94.2272 | 97.9723 |
| **Bahcecioglu et al. 2011** | 0.1776 | 0.0324 | 0.3879 | 67.5130 | 94.0752 | 89.0171 | 96.8038 |
| **Bal et al. 2021** | 0.2121 | 0.0383 | 0.4564 | 112.0169 | 96.4291 | 93.9330 | 97.8983 |
| **Dulger et al. 2014** | 0.1863 | 0.0000 | 0.5123 | 113.7277 | 96.4828 | 94.0390 | 97.9248 |
| **Serin et al.2019** | 0.2369 | 0.0177 | 0.5588 | 125.0748 | 96.8019 | 94.6619 | 98.0840 |
| **Yurtsever et al. 2011** | 0.3373 | 0.1350 | 0.5727 | 59.2376 | 93.2475 | 87.1789 | 96.4437 |

# Appendix Table 7. Random Effects Model Output for Outpatient Studies

| **Random effects model** |  |  |  |  |  |  |
| --- | --- | --- | --- | --- | --- | --- |
|  |  |  |  |  |  |  |
| **Study** | Prevalence | LCI 95% | HCI 95% | weight (%) | N | Cases |
| **Akgun et al 2016** | 0.0929 | 0.0497 | 0.1472 | 2.2432 | 140 | 13 |
| **Altinbas et al. 2012** | 0.0103 | 0.0013 | 0.0260 | 2.5603 | 291 | 3 |
| **Ayaz et al. 2013** | 0.0280 | 0.0243 | 0.0319 | 2.9305 | 7366 | 206 |
| **Ayaz et al. 2019** | 0.0444 | 0.0391 | 0.0500 | 2.9244 | 5471 | 243 |
| **Aydemir et al. 2019** | 0.0135 | 0.0063 | 0.0233 | 2.7822 | 740 | 10 |
| **Bahcecioglu et al. 2011** | 0.3869 | 0.3203 | 0.4557 | 2.4138 | 282 | 128 |
| **Bal et al. 2021** | 0.2601 | 0.2204 | 0.3019 | 2.6828 | 462 | 128 |
| **Berktas et al. 2012** | 0.0690 | 0.0326 | 0.1167 | 2.2617 | 145 | 10 |
| **Celen et al. 2006** | 0.0852 | 0.0688 | 0.1033 | 2.8245 | 1009 | 86 |
| **Celen et al. 2014** | 0.0590 | 0.0539 | 0.0643 | 2.9316 | 7871 | 464 |
| **Dagcioglu et al. 2019** | 0.0182 | 0.0003 | 0.0540 | 2.1064 | 110 | 2 |
| **Demiral et al. 2009** | 0.0279 | 0.0105 | 0.0525 | 2.5078 | 251 | 7 |
| **Dogan et al. 2013** | 0.0966 | 0.0769 | 0.1182 | 2.7915 | 787 | 76 |
| **Dulger et al. 2014** | 0.0859 | 0.0691 | 0.1043 | 2.8208 | 978 | 84 |
| **Duman et al. 2014** | 0.1510 | 0.1360 | 0.1666 | 2.8871 | 2093 | 316 |
| **Erdogan et al. 2012** | 0.0121 | 0.0015 | 0.0306 | 2.5018 | 247 | 3 |
| **Ergen et al. 2020** | 0.0290 | 0.0229 | 0.0359 | 2.8978 | 2548 | 74 |
| **Eryildiz et al. 2022** | 0.0360 | 0.0280 | 0.0451 | 2.8786 | 1831 | 66 |
| **Eser-Karlidag et al. 2019** | 0.0879 | 0.0635 | 0.1158 | 2.6875 | 455 | 40 |
| **Gurkan et al. 2013** | 0.0415 | 0.0334 | 0.0505 | 2.8879 | 2119 | 88 |
| **Inci et al. 2013** | 0.0344 | 0.0252 | 0.0448 | 2.8540 | 1339 | 46 |
| **Iskender et al. 2006** | 0.0233 | 0.0004 | 0.0688 | 1.9518 | 86 | 2 |
| **Karadag et al. 2014** | 0.0156 | 0.0109 | 0.0210 | 2.8928 | 2314 | 36 |
| **Kaya et al. 2006** | 0.0357 | 0.0077 | 0.0798 | 2.1172 | 112 | 4 |
| **Kaya et al. 2019** | 0.0239 | 0.0190 | 0.0295 | 2.9086 | 3257 | 78 |
| **Kolgelier et al. 2013** | 0.0325 | 0.0180 | 0.0508 | 2.6911 | 462 | 15 |
| **Korkmaz et al. 2014** | 0.0091 | 0.0026 | 0.0192 | 2.7280 | 547 | 5 |
| **Kose et al. 2012** | 0.0246 | 0.0194 | 0.0303 | 2.9066 | 3094 | 76 |
| **Kurt et al. 2021** | 0.0153 | 0.0101 | 0.0215 | 2.8786 | 1829 | 28 |
| **Parlak et al. 2015** | 0.0406 | 0.0332 | 0.0486 | 2.8977 | 2540 | 103 |
| **Sahin et al. 2018** | 0.0957 | 0.0725 | 0.1216 | 2.7306 | 554 | 53 |
| **Sarp et al. 2021** | 0.0713 | 0.0583 | 0.0856 | 2.8563 | 1374 | 98 |
| **Serin et al.2019** | 0.0175 | 0.0097 | 0.0275 | 2.8037 | 857 | 15 |
| **Yasar et al. 2011** | 0.0708 | 0.0528 | 0.0912 | 2.7713 | 692 | 49 |
| **Yolcu et al. 2018** | 0.0244 | 0.0182 | 0.0315 | 2.8870 | 2089 | 51 |
| **Yozgat et al. 2015** | 0.0300 | 0.0204 | 0.0413 | 2.8275 | 1035 | 31 |
| **Yurtsever et al. 2011** | 0.0636 | 0.0467 | 0.0828 | 2.7751 | 708 | 45 |
|  |  |  |  |  |  |  |
| **Pooled** | 0.050595859 | 0.040038245 | 0.06230603 | 100 |  |  |
| **Statistics** |  |  |  |  |  |  |
| **I-squared** | 97.1554376 | 96.639845 | 97.5919161 |  |  |  |
| **Cochran's Q** | 1265.57252 |  |  |  |  |  |
| **Chi2, p** | 0 |  |  |  |  |  |
| **tau2** | 0.022650456 |  |  |  |  |  |

#

# Appendix Table 8. Random Effects Model Sensitivity for Outpatient Studies

| **Random effects sensitivity** |  |  |  |  |  |  |  |
| --- | --- | --- | --- | --- | --- | --- | --- |
|  |  |  |  |  |  |  |  |
| **Study** | Pooled Prevalence | LCI 95% | HCI 95% | Cochran Q | I 2 | I 2 LCI 95% | I 2 HCI 95% |
| **Akgun et al 2016** | 0.0502 | 0.0397 | 0.0618 | 1262.2382 | 97.1479 | 96.6305 | 97.5859 |
| **Altinbas et al. 2012** | 0.0524 | 0.0417 | 0.0643 | 1257.3001 | 97.1367 | 96.6166 | 97.5769 |
| **Ayaz et al. 2013** | 0.0519 | 0.0407 | 0.0643 | 1215.6716 | 97.0387 | 96.4944 | 97.4985 |
| **Ayaz et al. 2019** | 0.0513 | 0.0401 | 0.0637 | 1268.1175 | 97.1611 | 96.6469 | 97.5965 |
| **Aydemir et al. 2019** | 0.0524 | 0.0417 | 0.0643 | 1245.1239 | 97.1087 | 96.5817 | 97.5545 |
| **Bahcecioglu et al. 2011** | 0.0465 | 0.0370 | 0.0569 | 1096.1933 | 96.7159 | 96.0903 | 97.2414 |
| **Bal et al. 2021** | 0.0474 | 0.0379 | 0.0579 | 1075.0909 | 96.6514 | 96.0092 | 97.1903 |
| **Berktas et al. 2012** | 0.0506 | 0.0401 | 0.0623 | 1266.2928 | 97.1571 | 96.6419 | 97.5932 |
| **Celen et al. 2006** | 0.0502 | 0.0397 | 0.0618 | 1235.9907 | 97.0874 | 96.5551 | 97.5374 |
| **Celen et al. 2014** | 0.0509 | 0.0398 | 0.0632 | 1217.3816 | 97.0428 | 96.4996 | 97.5018 |
| **Dagcioglu et al. 2019** | 0.0518 | 0.0411 | 0.0636 | 1267.1644 | 97.1590 | 96.6443 | 97.5948 |
| **Demiral et al. 2009** | 0.0517 | 0.0410 | 0.0635 | 1267.4452 | 97.1596 | 96.6451 | 97.5953 |
| **Dogan et al. 2013** | 0.0500 | 0.0395 | 0.0615 | 1230.7066 | 97.0749 | 96.5395 | 97.5274 |
| **Dulger et al. 2014** | 0.0502 | 0.0397 | 0.0618 | 1236.1582 | 97.0878 | 96.5556 | 97.5377 |
| **Duman et al. 2014** | 0.0487 | 0.0395 | 0.0589 | 950.2353 | 96.2115 | 95.4533 | 96.8432 |
| **Erdogan et al. 2012** | 0.0523 | 0.0415 | 0.0641 | 1260.7645 | 97.1446 | 96.6263 | 97.5832 |
| **Ergen et al. 2020** | 0.0518 | 0.0409 | 0.0639 | 1254.8039 | 97.1310 | 96.6095 | 97.5724 |
| **Eryildiz et al. 2022** | 0.0515 | 0.0407 | 0.0636 | 1266.6789 | 97.1579 | 96.6429 | 97.5939 |
| **Eser-Karlidag et al. 2019** | 0.0502 | 0.0397 | 0.0618 | 1252.1588 | 97.1250 | 96.6019 | 97.5675 |
| **Gurkan et al. 2013** | 0.0514 | 0.0405 | 0.0634 | 1268.6136 | 97.1623 | 96.6483 | 97.5974 |
| **Inci et al. 2013** | 0.0516 | 0.0408 | 0.0636 | 1266.3541 | 97.1572 | 96.6420 | 97.5933 |
| **Iskender et al. 2006** | 0.0516 | 0.0409 | 0.0633 | 1268.1420 | 97.1612 | 96.6470 | 97.5965 |
| **Karadag et al. 2014** | 0.0524 | 0.0417 | 0.0642 | 1203.9045 | 97.0097 | 96.4583 | 97.4753 |
| **Kaya et al. 2006** | 0.0513 | 0.0407 | 0.0631 | 1268.6280 | 97.1623 | 96.6484 | 97.5974 |
| **Kaya et al. 2019** | 0.0520 | 0.0411 | 0.0641 | 1231.3443 | 97.0764 | 96.5414 | 97.5286 |
| **Kolgelier et al. 2013** | 0.0516 | 0.0409 | 0.0635 | 1267.5898 | 97.1600 | 96.6455 | 97.5955 |
| **Korkmaz et al. 2014** | 0.0526 | 0.0419 | 0.0645 | 1243.0575 | 97.1039 | 96.5757 | 97.5506 |
| **Kose et al. 2012** | 0.0520 | 0.0411 | 0.0640 | 1236.0057 | 97.0874 | 96.5551 | 97.5374 |
| **Kurt et al. 2021** | 0.0524 | 0.0417 | 0.0642 | 1216.9437 | 97.0418 | 96.4982 | 97.5009 |
| **Parlak et al. 2015** | 0.0514 | 0.0405 | 0.0635 | 1268.4242 | 97.1618 | 96.6478 | 97.5970 |
| **Sahin et al. 2018** | 0.0500 | 0.0395 | 0.0616 | 1242.6048 | 97.1029 | 96.5744 | 97.5498 |
| **Sarp et al. 2021** | 0.0505 | 0.0399 | 0.0623 | 1246.2247 | 97.1113 | 96.5849 | 97.5565 |
| **Serin et al.2019** | 0.0522 | 0.0415 | 0.0641 | 1250.0767 | 97.1202 | 96.5960 | 97.5637 |
| **Yasar et al. 2011** | 0.0505 | 0.0400 | 0.0623 | 1257.6295 | 97.1375 | 96.6175 | 97.5775 |
| **Yolcu et al. 2018** | 0.0520 | 0.0411 | 0.0640 | 1246.7890 | 97.1126 | 96.5865 | 97.5576 |
| **Yozgat et al. 2015** | 0.0517 | 0.0410 | 0.0637 | 1264.1751 | 97.1523 | 96.6359 | 97.5894 |
| **Yurtsever et al. 2011** | 0.0507 | 0.0401 | 0.0625 | 1261.9665 | 97.1473 | 96.6297 | 97.5854 |

# Appendix Table 9. Quality Effects Model Output for Outpatient Studies

| **Quality effects model** |  |  |  |  |  |  |
| --- | --- | --- | --- | --- | --- | --- |
|  |  |  |  |  |  |  |
| **Study** | Prevalence | LCI 95% | HCI 95% | weight (%) | N | Cases |
| **Akgun et al 2016** | 0.0929 | 0.0497 | 0.1472 | 0.8050 | 140 | 13 |
| **Altinbas et al. 2012** | 0.0103 | 0.0013 | 0.0260 | 0.9873 | 291 | 3 |
| **Ayaz et al. 2013** | 0.0280 | 0.0243 | 0.0319 | 13.6077 | 7366 | 206 |
| **Ayaz et al. 2019** | 0.0444 | 0.0391 | 0.0500 | 7.2385 | 5471 | 243 |
| **Aydemir et al. 2019** | 0.0135 | 0.0063 | 0.0233 | 1.3107 | 740 | 10 |
| **Bahcecioglu et al. 2011** | 0.3869 | 0.3203 | 0.4557 | 0.7511 | 282 | 128 |
| **Bal et al. 2021** | 0.2601 | 0.2204 | 0.3019 | 1.1743 | 462 | 128 |
| **Berktas et al. 2012** | 0.0690 | 0.0326 | 0.1167 | 0.6952 | 145 | 10 |
| **Celen et al. 2006** | 0.0852 | 0.0688 | 0.1033 | 2.6482 | 1009 | 86 |
| **Celen et al. 2014** | 0.0590 | 0.0539 | 0.0643 | 14.4783 | 7871 | 464 |
| **Dagcioglu et al. 2019** | 0.0182 | 0.0003 | 0.0540 | 0.6590 | 110 | 2 |
| **Demiral et al. 2009** | 0.0279 | 0.0105 | 0.0525 | 0.9390 | 251 | 7 |
| **Dogan et al. 2013** | 0.0966 | 0.0769 | 0.1182 | 1.3593 | 787 | 76 |
| **Dulger et al. 2014** | 0.0859 | 0.0691 | 0.1043 | 1.5569 | 978 | 84 |
| **Duman et al. 2014** | 0.1510 | 0.1360 | 0.1666 | 3.1619 | 2093 | 316 |
| **Erdogan et al. 2012** | 0.0121 | 0.0015 | 0.0306 | 1.2011 | 247 | 3 |
| **Ergen et al. 2020** | 0.0290 | 0.0229 | 0.0359 | 3.1809 | 2548 | 74 |
| **Eryildiz et al. 2022** | 0.0360 | 0.0280 | 0.0451 | 4.0654 | 1831 | 66 |
| **Eser-Karlidag et al. 2019** | 0.0879 | 0.0635 | 0.1158 | 1.1852 | 455 | 40 |
| **Gurkan et al. 2013** | 0.0415 | 0.0334 | 0.0505 | 2.7371 | 2119 | 88 |
| **Inci et al. 2013** | 0.0344 | 0.0252 | 0.0448 | 1.9303 | 1339 | 46 |
| **Iskender et al. 2006** | 0.0233 | 0.0004 | 0.0688 | 0.5285 | 86 | 2 |
| **Karadag et al. 2014** | 0.0156 | 0.0109 | 0.0210 | 3.4286 | 2314 | 36 |
| **Kaya et al. 2006** | 0.0357 | 0.0077 | 0.0798 | 0.7713 | 112 | 4 |
| **Kaya et al. 2019** | 0.0239 | 0.0190 | 0.0295 | 4.5667 | 3257 | 78 |
| **Kolgelier et al. 2013** | 0.0325 | 0.0180 | 0.0508 | 1.0231 | 462 | 15 |
| **Korkmaz et al. 2014** | 0.0091 | 0.0026 | 0.0192 | 1.2962 | 547 | 5 |
| **Kose et al. 2012** | 0.0246 | 0.0194 | 0.0303 | 3.7457 | 3094 | 76 |
| **Kurt et al. 2021** | 0.0153 | 0.0101 | 0.0215 | 2.8433 | 1829 | 28 |
| **Parlak et al. 2015** | 0.0406 | 0.0332 | 0.0486 | 3.7014 | 2540 | 103 |
| **Sahin et al. 2018** | 0.0957 | 0.0725 | 0.1216 | 1.1183 | 554 | 53 |
| **Sarp et al. 2021** | 0.0713 | 0.0583 | 0.0856 | 2.2942 | 1374 | 98 |
| **Serin et al.2019** | 0.0175 | 0.0097 | 0.0275 | 1.6703 | 857 | 15 |
| **Yasar et al. 2011** | 0.0708 | 0.0528 | 0.0912 | 1.4712 | 692 | 49 |
| **Yolcu et al. 2018** | 0.0244 | 0.0182 | 0.0315 | 2.7061 | 2089 | 51 |
| **Yozgat et al. 2015** | 0.0300 | 0.0204 | 0.0413 | 1.8851 | 1035 | 31 |
| **Yurtsever et al. 2011** | 0.0636 | 0.0467 | 0.0828 | 1.2776 | 708 | 45 |
|  |  |  |  |  |  |  |
| **Pooled** | 0.043903772 | 0.02996631 | 0.06030452 | 100 |  |  |
| **Statistics** |  |  |  |  |  |  |
| **I-squared** | 97.1554376 | 96.639845 | 97.5919161 |  |  |  |
| **Cochran's Q** | 1265.57252 |  |  |  |  |  |
| **Chi2, p** | 0 |  |  |  |  |  |
| **Q-Index** | 23.33155186 |  |  |  |  |  |

# Appendix Table 10. Quality Effects Model Sensitivity Output for Outpatient Studies

| **Quality effects sensitivity** |  |  |  |  |  |  |  |
| --- | --- | --- | --- | --- | --- | --- | --- |
|  |  |  |  |  |  |  |  |
| **Excluded study** | Pooled Prevalence | LCI 95% | HCI 95% | Cochran Q | I 2 | I 2 LCI 95% | I 2 HCI 95% |
| **Akgun et al 2016** | 0.0438 | 0.0299 | 0.0602 | 1262.2382 | 97.1479 | 96.6305 | 97.5859 |
| **Altinbas et al. 2012** | 0.0446 | 0.0306 | 0.0611 | 1257.3001 | 97.1367 | 96.6166 | 97.5769 |
| **Ayaz et al. 2013** | 0.0471 | 0.0327 | 0.0640 | 1215.6716 | 97.0387 | 96.4944 | 97.4985 |
| **Ayaz et al. 2019** | 0.0439 | 0.0288 | 0.0619 | 1268.1175 | 97.1611 | 96.6469 | 97.5965 |
| **Aydemir et al. 2019** | 0.0446 | 0.0305 | 0.0612 | 1245.1239 | 97.1087 | 96.5817 | 97.5545 |
| **Bahcecioglu et al. 2011** | 0.0428 | 0.0300 | 0.0578 | 1096.1933 | 96.7159 | 96.0903 | 97.2414 |
| **Bal et al. 2021** | 0.0427 | 0.0299 | 0.0576 | 1075.0909 | 96.6514 | 96.0092 | 97.1903 |
| **Berktas et al. 2012** | 0.0440 | 0.0301 | 0.0604 | 1266.2928 | 97.1571 | 96.6419 | 97.5932 |
| **Celen et al. 2006** | 0.0432 | 0.0293 | 0.0596 | 1235.9907 | 97.0874 | 96.5551 | 97.5374 |
| **Celen et al. 2014** | 0.0420 | 0.0286 | 0.0579 | 1217.3816 | 97.0428 | 96.4996 | 97.5018 |
| **Dagcioglu et al. 2019** | 0.0443 | 0.0303 | 0.0608 | 1267.1644 | 97.1590 | 96.6443 | 97.5948 |
| **Demiral et al. 2009** | 0.0443 | 0.0303 | 0.0608 | 1267.4452 | 97.1596 | 96.6451 | 97.5953 |
| **Dogan et al. 2013** | 0.0435 | 0.0297 | 0.0598 | 1230.7066 | 97.0749 | 96.5395 | 97.5274 |
| **Dulger et al. 2014** | 0.0436 | 0.0296 | 0.0600 | 1236.1582 | 97.0878 | 96.5556 | 97.5377 |
| **Duman et al. 2014** | 0.0418 | 0.0295 | 0.0560 | 950.2353 | 96.2115 | 95.4533 | 96.8432 |
| **Erdogan et al. 2012** | 0.0446 | 0.0306 | 0.0612 | 1260.7645 | 97.1446 | 96.6263 | 97.5832 |
| **Ergen et al. 2020** | 0.0445 | 0.0299 | 0.0618 | 1254.8039 | 97.1310 | 96.6095 | 97.5724 |
| **Eryildiz et al. 2022** | 0.0445 | 0.0300 | 0.0616 | 1266.6789 | 97.1579 | 96.6429 | 97.5939 |
| **Eser-Karlidag et al. 2019** | 0.0437 | 0.0298 | 0.0601 | 1252.1588 | 97.1250 | 96.6019 | 97.5675 |
| **Gurkan et al. 2013** | 0.0441 | 0.0296 | 0.0613 | 1268.6136 | 97.1623 | 96.6483 | 97.5974 |
| **Inci et al. 2013** | 0.0443 | 0.0300 | 0.0612 | 1266.3541 | 97.1572 | 96.6420 | 97.5933 |
| **Iskender et al. 2006** | 0.0442 | 0.0303 | 0.0606 | 1268.1420 | 97.1612 | 96.6470 | 97.5965 |
| **Karadag et al. 2014** | 0.0453 | 0.0309 | 0.0623 | 1203.9045 | 97.0097 | 96.4583 | 97.4753 |
| **Kaya et al. 2006** | 0.0442 | 0.0302 | 0.0606 | 1268.6280 | 97.1623 | 96.6484 | 97.5974 |
| **Kaya et al. 2019** | 0.0451 | 0.0304 | 0.0625 | 1231.3443 | 97.0764 | 96.5414 | 97.5286 |
| **Kolgelier et al. 2013** | 0.0443 | 0.0302 | 0.0608 | 1267.5898 | 97.1600 | 96.6455 | 97.5955 |
| **Korkmaz et al. 2014** | 0.0448 | 0.0307 | 0.0613 | 1243.0575 | 97.1039 | 96.5757 | 97.5506 |
| **Kose et al. 2012** | 0.0448 | 0.0302 | 0.0622 | 1236.0057 | 97.0874 | 96.5551 | 97.5374 |
| **Kurt et al. 2021** | 0.0451 | 0.0308 | 0.0620 | 1216.9437 | 97.0418 | 96.4982 | 97.5009 |
| **Parlak et al. 2015** | 0.0442 | 0.0296 | 0.0615 | 1268.4242 | 97.1618 | 96.6478 | 97.5970 |
| **Sahin et al. 2018** | 0.0437 | 0.0298 | 0.0600 | 1242.6048 | 97.1029 | 96.5744 | 97.5498 |
| **Sarp et al. 2021** | 0.0435 | 0.0294 | 0.0602 | 1246.2247 | 97.1113 | 96.5849 | 97.5565 |
| **Serin et al.2019** | 0.0447 | 0.0305 | 0.0613 | 1250.0767 | 97.1202 | 96.5960 | 97.5637 |
| **Yasar et al. 2011** | 0.0438 | 0.0298 | 0.0603 | 1257.6295 | 97.1375 | 96.6175 | 97.5775 |
| **Yolcu et al. 2018** | 0.0447 | 0.0302 | 0.0617 | 1246.7890 | 97.1126 | 96.5865 | 97.5576 |
| **Yozgat et al. 2015** | 0.0444 | 0.0302 | 0.0612 | 1264.1751 | 97.1523 | 96.6359 | 97.5894 |
| **Yurtsever et al. 2011** | 0.0439 | 0.0298 | 0.0604 | 1261.9665 | 97.1473 | 96.6297 | 97.5854 |

# Appendix Table 11. Random Effects Model Output for Blood Donor and Serosurvey Studies

| **Random effects results** |  |  |  |  |  |  |  |
| --- | --- | --- | --- | --- | --- | --- | --- |
|  |  |  |  |  |  |  |  |
| **Study** | Prevalence | LCI 95% | HCI 95% | weight (%) | N | HBsAg-positive | delta cases |
| **Mese et al. 2014** | 0.069892473 | 0.037131932 | 0.111520814 | 26.29070184 | 6200 | 186 | 13 |
| **Sirmatel et al. 2012** | 0.02293578 | 0.006467956 | 0.047863184 | 28.2970245 | 21262 | 218 | 5 |
| **Uzun et al. 2014** | 0.034090909 | 0.004312958 | 0.0846445 | 17.11524915 | 12423 | 88 | 3 |
| **Tozun et al. 2014** | 0.027522936 | 0.009223409 | 0.054177195 | 28.2970245 | 5460 | 218 | 6 |
|  |  |  |  |  |  |  |  |
| **Pooled** | 0.037960169 | 0.019984261 | 0.061105898 | 100 |  |  |  |
| **Statistics** |  |  |  |  |  |  |  |
| **I-squared** | 49.66285553 | 0 | 83.34471249 |  |  |  |  |
| **Cochran's Q** | 5.959813636 |  |  |  |  |  |  |
| **Chi2, p** | 0.11358184 |  |  |  |  |  |  |
| **tau2** | 0.005713477 |  |  |  |  |  |  |

# Appendix Table 12. Sensitivity Analysis per Study for Blood Donor and Serosurvey Studies

| **Random effects sensitivity** |  |  |  |  |  |  |  |  |
| --- | --- | --- | --- | --- | --- | --- | --- | --- |
|  |  |  |  |  |  |  |  |  |
| **Study** | Pooled Prevalence | LCI 95% | HCI 95% | Cochran Q | p | I 2 | I 2 LCI 95% | I 2 HCI 95% |
| **Mese et al. 2014** | 0.028263617 | 0.015599181 | 0.04437763 | 0.409223871 | 0.81496351 | 0 | 0 | 49.1622694 |
| **Sirmatel et al. 2012** | 0.04377684 | 0.020513432 | 0.074707876 | 4.052009157 | 0.13186131 | 50.64176999 | 0 | 85.7373673 |
| **Uzun et al. 2014** | 0.037322868 | 0.015148201 | 0.068034274 | 5.959179785 | 0.05081367 | 66.43833426 | 0 | 90.3258431 |
| **Tozun et al. 2014** | 0.040991076 | 0.015963233 | 0.075951371 | 5.180369521 | 0.07500618 | 61.39271548 | 0 | 88.9900859 |

# Appendix Table 13. Quality Effects Model Output for Blood Donor and Serosurvey Studies

| **Quality effects model results** |  |  |  |  |  |  |  |
| --- | --- | --- | --- | --- | --- | --- | --- |
|  |  |  |  |  |  |  |  |
| **Study** | Prevalence | LCI 95% | HCI 95% | weight (%) | N | HBsAg-positive | delta cases |
| **Mese et al. 2014** | 0.069892473 | 0.037131932 | 0.111520814 | 24.178911 | 6200 | 186 | 13 |
| **Sirmatel et al. 2012** | 0.02293578 | 0.006467956 | 0.047863184 | 31.359839 | 21262 | 218 | 5 |
| **Uzun et al. 2014** | 0.034090909 | 0.004312958 | 0.0846445 | 13.101412 | 12423 | 88 | 3 |
| **Tozun et al. 2014** | 0.027522936 | 0.009223409 | 0.054177195 | 31.359839 | 5460 | 218 | 6 |
|  |  |  |  |  |  |  |  |
| **Pooled** | 0.036558607 | 0.018807443 | 0.059587946 | 100 |  |  |  |
| **Statistics** |  |  |  |  |  |  |  |
| **I-squared** | 49.66285553 | 0 | 83.34471249 |  |  |  |  |
| **Cochran's Q** | 5.959813636 |  |  |  |  |  |  |
| **Chi2, p** | 0.11358184 |  |  |  |  |  |  |
| **Q-Index** | 2.619382022 |  |  |  |  |  |  |

# Appendix Table 14. Quality Effects Model Blood Donor and Serosurvey Studies Sensitivity

| **Quality effects sensitivity** |  |  |  |  |  |  |  |  |
| --- | --- | --- | --- | --- | --- | --- | --- | --- |
|  |  |  |  |  |  |  |  |  |
| **study** | Pooled Prevalence | LCI 95% | HCI 95% | Cochran Q | p | I 2 | I 2 LCI 95% | I 2 HCI 95% |
| **Mese et al. 2014** | 0.028263617 | 0.01559918 | 0.04437763 | 0.409223871 | 0.814963513 | 0 | 0 | 49.16226941 |
| **Sirmatel et al. 2012** | 0.042621946 | 0.01947371 | 0.073629888 | 4.052009157 | 0.131861312 | 50.64176999 | 0 | 85.73736733 |
| **Uzun et al. 2014** | 0.03554464 | 0.01388899 | 0.065846 | 5.959179785 | 0.050813669 | 66.43833426 | 0 | 90.32584313 |
| **Tozun et al. 2014** | 0.039413489 | 0.01458014 | 0.074555185 | 5.180369521 | 0.075006181 | 61.39271548 | 0 | 88.99008593 |

# Appendix Figure 1: DOI plot for the inpatient studies to assess the impact of individual studies on the overall estimate of effect size

#
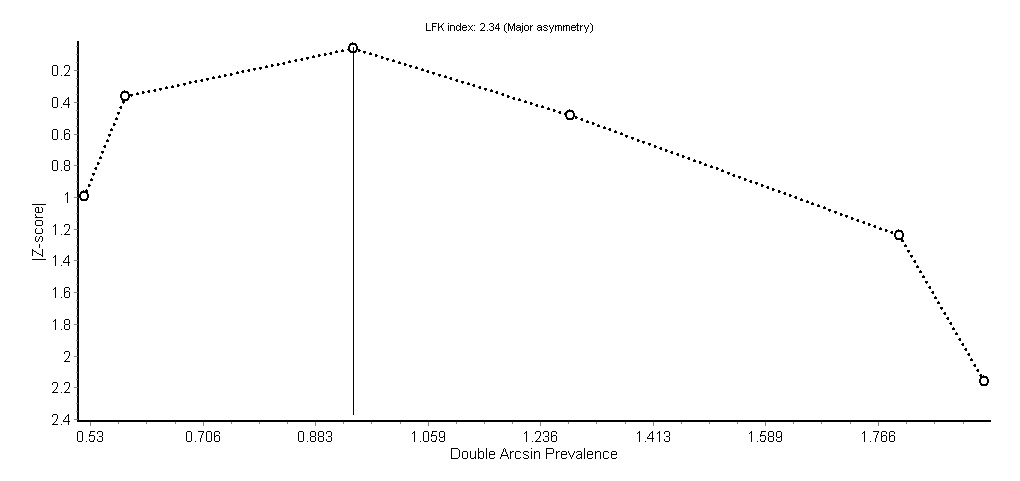


The plot displays the standardized effect size for each study on the x-axis and the inverse of the standard error of the effects size on the y-axis. A horizontal line is drawn at the overall effect size estimate. Each study is represented by a point on the plot, with the size of the point corresponding to the weight of the study in the meta-analysis. The plot also includes a regression line and two diagonal lines representing the 95% confidence intervals for the regression line.

Appendix Figure 2: Funnel plot for the inpatient studies to assess the presence of publication bias


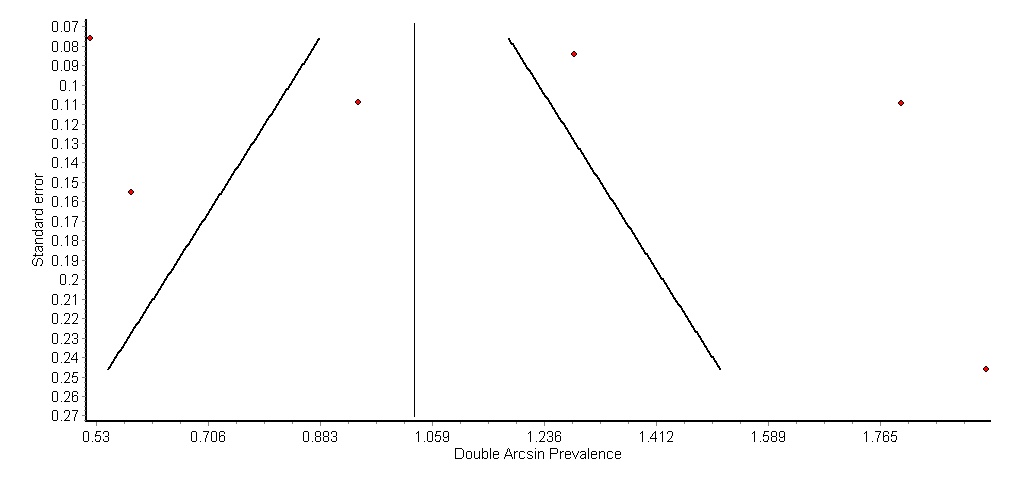


The plot shows the effect size of each study on the x-axis and a measure of the precision of the effect size estimate on the y-axis.

# Appendix Figure 3: DOI plot for the outpatient studies to assess the impact of individual studies on the overall estimate of effect size


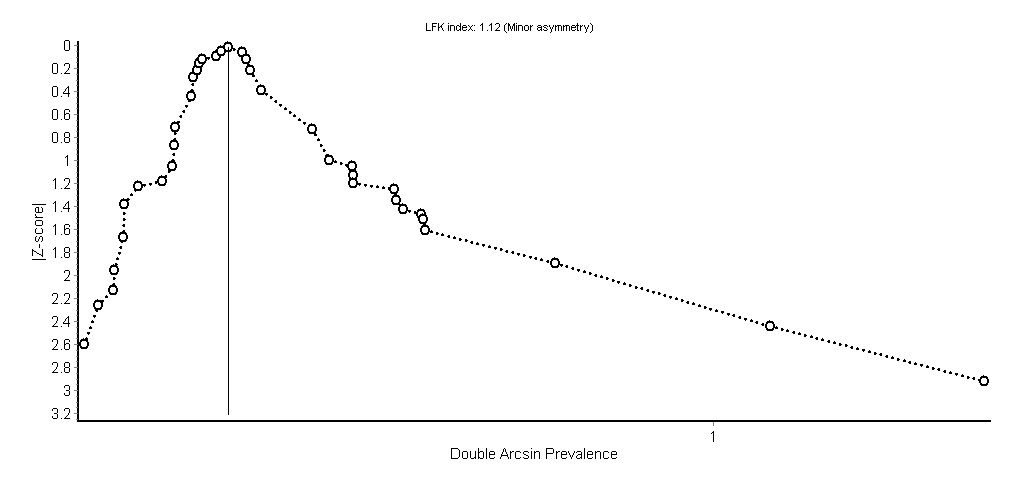


The plot displays the standardized effect size for each study on the x-axis and the inverse of the standard error of the effects size on the y-axis. A horizontal line is drawn at the overall effect size estimate. Each study is represented by a point on the plot, with the size of the point corresponding to the weight of the study in the meta-analysis. The plot also includes a regression line and two diagonal lines representing the 95% confidence intervals for the regression line.

# Appendix Figure 2: Funnel plot for the outpatient studies to assess the presence of publication bias


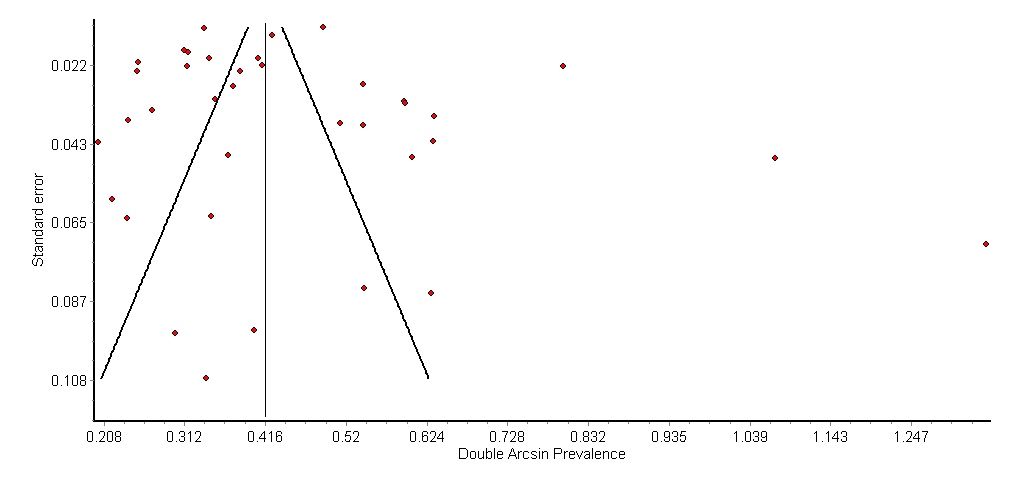


The plot shows the effect size of each study on the x-axis and a measure of the precision of the effect size estimate on the y-axis.

# Appendix Figure 5: DOI plot for the blood donor and serosurvey studies to assess the impact of individual studies on the overall estimate of effect size


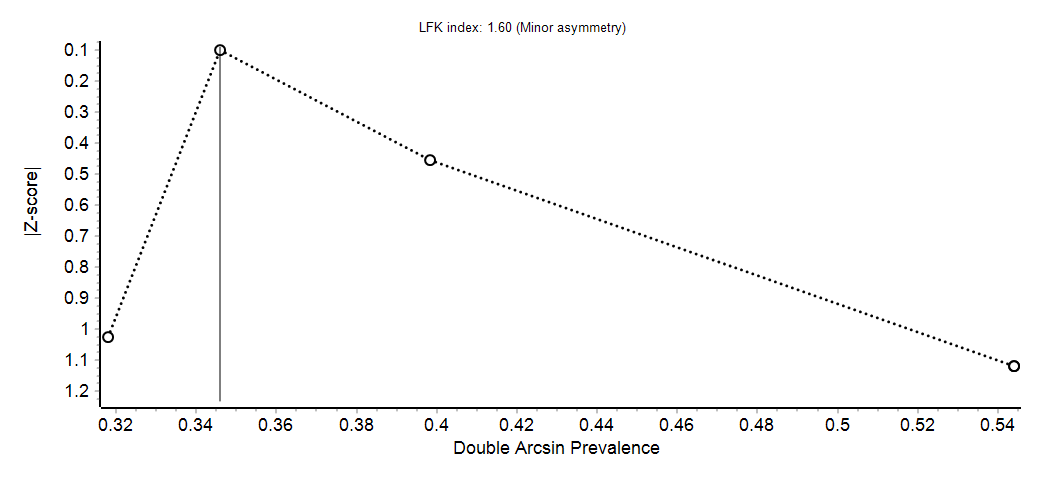


The plot displays the standardized effect size for each study on the x-axis and the inverse of the standard error of the effects size on the y-axis. A horizontal line is drawn at the overall effect size estimate. Each study is represented by a point on the plot, with the size of the point corresponding to the weight of the study in the meta-analysis. The plot also includes a regression line and two diagonal lines representing the 95% confidence intervals for the regression line.

# Appendix Figure 6: Funnel plot for the blood donor and serosurvey studies to assess the presence of publication bias


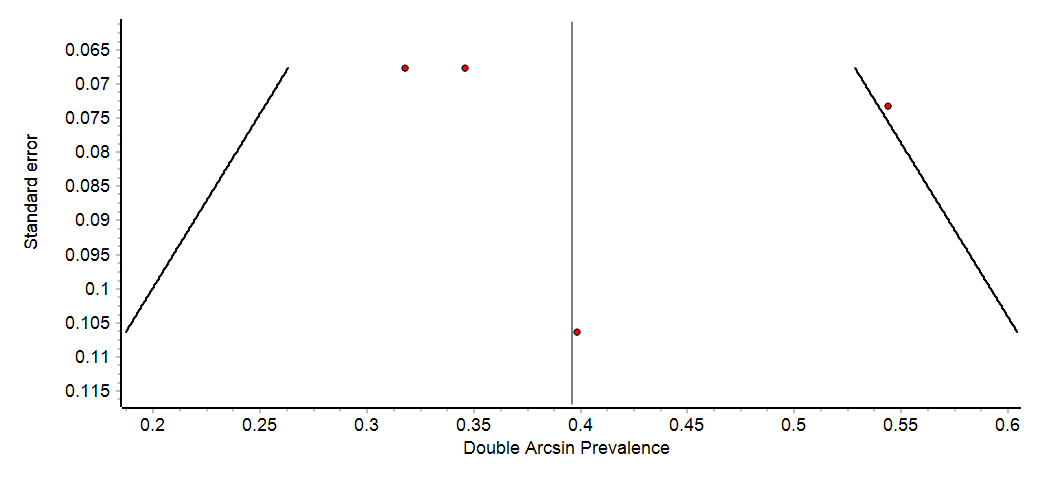


The plot shows the effect size of each study on the x-axis and a measure of the precision of the effect size estimate on the y-axis.
